# Vitamin D Receptor Agonists Target CXCL10: New Therapeutic Tools for Resolution of Inflammation

**DOI:** 10.1155/2013/876319

**Published:** 2013-04-17

**Authors:** Sabino Scolletta, Marta Colletti, Luigi Di Luigi, Clara Crescioli

**Affiliations:** ^1^Department of Medical Biotechnologies, University of Siena, Viale Bracci 1, 53100 Siena, Italy; ^2^Department of Movement, Human and Health Sciences, Unit of Endocrinology, University of Rome Foro Italico, Piazza Lauro de Bosis 15, 00135 Rome, Italy

## Abstract

Understanding the many biological extraskeletal actions of vitamin D has increased in the past decades. Indeed, vitamin D and analogue molecules, besides the classical actions on bone metabolism, exert several beneficial effects on metabolic homeostasis, heart-cardiovascular, brain, and muscle physiological functions, throughout the interaction with the specific vitamin D receptor (VDR). In particular, VDR agonists powerfully control innate and adaptive immune system with favorable effects on human health. VDR ligands act as immunomodulators that are potent enough to retain anti-inflammatory effects, even though the mechanism underlying those effects is not yet fully elucidated. VDR agonists exert a significant suppression of inflammatory processes switching the immune response from T helper 1 (Th1) to T helper 2 (Th2) dominance and counteracting the self-enhancing inflammatory loop between immune and resident cells, especially by cytokine release impairment. Those molecules are able, indeed, to reduce the release of the interferon (IFN)*γ*-induced 10 kDa protein IP-10/CXCL10, a powerful chemokine driving Th1-mediated inflammation. Based on their features, VDR ligands show the potentiality to be included in immunosuppressive regimens, aimed to control auto- and alloimmune Th1-driven overreactivity, occurring, for example, in autoimmune disease or graft rejection.

## 1. Introduction

The concept that vitamin D, classically categorized as a regulator of calcium/phosphorous balance and bone metabolism, is able to act on the immune system has emerged more than 20 years ago [1, 2]. Since then, accumulating evidence confirmed that those pharmacologic effects on the immune system are suppressive enough to retain therapeutic potentials for the management of immune-related inflammatory diseases. Furthermore, epidemiological studies show that circulating altered levels of vitamin D are associated with a higher susceptibility to immune-mediated disorders and inflammatory diseases [3].

The pleiotropic activities in immune regulation by VDR agonists rely on their ability to interfere with maturation/differentiation/activation of the majority of the immune system cells which express VDR, such as monocytes, macrophages, B and T lymphocytes, neutrophils, and dendritic cells (DCs). Vitamin D, for example, is able to suppress cellular immune response by inhibiting the proliferation of T cells and the maturation of DCs, the most potent antigen-presenting cells (APCs) [4]. Hence, vitamin D can polarize Th1 immune response, which dominates in inflammation, toward a more regulatory Th2 phenotype, which dominates in tolerogenicity, by specifically repressing Th1 cytokine gene transcription in immune cells. Notably, VDR agonists are able to inhibit cytokine expression and release also in tissue resident cells, with a definite anti-inflammatory effect. 

In particular, we have previously reported on the ability of two less or nonhypercalcemic VDR ligands, BXL-01-0029 and elocalcitol, to counteract in lymphocytes and different human resident cell types the release of CXCL10 [5–7]; this is a key chemokine triggering Th1 inflammatory molecular processes, in auto- or alloimmune response, secreted by several types of resident cells (skeletal muscle cells, thyrocytes, cardiomyocytes, tubular renal cells, and human adrenal cells) [7–12]. 

In the present paper we intend to offer an overview on VDR agonists as new pharmacological tools with anti-inflammatory properties on immune and tissue resident cells, with particular attention to CXCL10, as a new biomolecular target for resolution of inflammation. 

## 2. Vitamin D

The pleiotropic hormone vitamin D, also known as vitamin D_3_ or calcitriol, is known since almost 90 years ago to prevent rickets in children, osteomalacia in adults, and hypocalcemic tetany [13, 14]; for long time, its function has been considered to be exerted exclusively on calcium, phosphorus, and bone metabolism. With the years, beside the other vitamin D functions—for example, on metabolism, cardiovascular system, muscle and brain functions, and cell growth/differentiation—important effects have been documented on the immune system. In fact, more than 25 years ago, the immunomodulatory role of vitamin D emerged [3] after the observation that monocytes/macrophages from patients affected by granulomatous disease sarcoidosis constitutively synthesize the active form of vitamin D [4]. Vitamin D is synthesized through a multistep process, which begins in the skin. The ultraviolet light (appropriate wavelength: 270–300 nm) photocatalyzes the conversion of the precursor 7-dehydrocholesterol to previtamin D_3_ or cholecalciferol, without any significant biological activity until its conversion to the hormonally active form, 1,25-dihydroxycholecalciferol. This conversion occurs in two steps. Within the liver, cholecalciferol is hydroxylated to 25-hydroxycholecalciferol [25(OH)D_3_] by the enzyme 25-hydroxylase (CYP2R1); within the kidney, 25-hydroxycholecalciferol serves as a substrate for 1-alpha-hydroxylase (CYP27B1), yielding 1,25-dihydroxycholecalciferol or calcitriol [1,25(OH)_2_D_3_], the biologically active form. Each form of vitamin D is hydrophobic and transported throughout the body by the specific vitamin D binding proteins (DBP). Vitamin D action is limited by catabolism—mainly by a 24-hydroxylase (CYP24A1)—which results in 1,24,25-trihydroxyvitamin D_3_ [1,24,25(OH)_3_D_3_], a compound with substantially lower affinity for the VDR; this compound is further metabolized to calcitroic acid and secreted in urine. The metabolism of vitamin D is complex and tightly regulated [15]. Rate limiting steps in the metabolism of vitamin D are the activity of CYP2R1, induced by low 25(OH)D_3_ levels, and the activity of CYP24A1, induced by high levels of 25(OH)D_3_, and 1,25(OH)D_3_ to avoid vitamin D toxicity. While liver and kidney are the main sites for vitamin D synthesis and degradation, many other tissues (colon, prostate, breast, lung, pancreas, brain, and endothelium) can synthesize and degrade the active form of vitamin D. Vitamin D biologic effects are exerted throughout the interaction with VDR, which is known to be present in over 30 human target tissues, as reported in Table 1 [16, 17]. 

Such a diffuse expression in several different tissues, together with metabolic enzyme presence, suggests paracrine/autocrine mechanisms of actions, in addition to the classical endocrine effect of the hormone. 

The dominant genomic pathway, by which vitamin D mediates its biologic effects, involves the regulation of target genes by ligand-receptor complex in the nucleus of target cells [18]. Summarizing, upon ligand-nuclear VDR interaction, vitamin D forms heterodimers with the retinoid X receptor (RXR) and its ligand (9 cis-retinoic acid); these dimers subsequently occupy specific binding sites on DNA, the vitamin D response elements (VDREs). In conjunction with other transcription factors, this complex induces the transcription of vitamin D responsive genes [19, 20]. Besides the well-characterized nuclear VDR, a less clearly defined cell membrane receptor, which mediates rapid nongenomic actions, has been hypothesized [21, 22]. Rapid nongenomic actions of vitamin D do not affect the nuclear transcriptional activity. Even if those vitamin D rapid mechanisms are still unclear, evidence suggests that the initiation of the fast nongenomic signal may involve the engagement of either a novel membrane receptor [23] or the nuclear VDR translocation to the cell surface [24].

Vitamin D classical genomic and “new” nongenomic actions are involved in the regulation of several critical functions, such as immunity, angiogenesis, differentiation, apoptosis, and cell growth. The inhibition, for example, by VDR ligands of prostate cell growth, either growth factor induced or neoplastic, is exerted throughout a rapid mechanism that blocks the phosphorylation/activation of growth factor receptors [25, 26]. In particular, due to their antiproliferative and prodifferentiation properties, known since quite ago [27], VDR ligands control tumoral cell growth in different models of cancer, such as prostate, breast, and colon [28–30]. Moreover, a strong epidemiological association between prostate, breast, colon cancer, and vitamin D deficiency has been documented [31].

Interestingly, vitamin D plays a pivotal role also in immune system cell control and differentiation, with important effects on the immune-mediated response. 

## 3. Protolerogenic Effects of VDR Agonists

### 3.1. Immune System Cells

**Figure 1:**
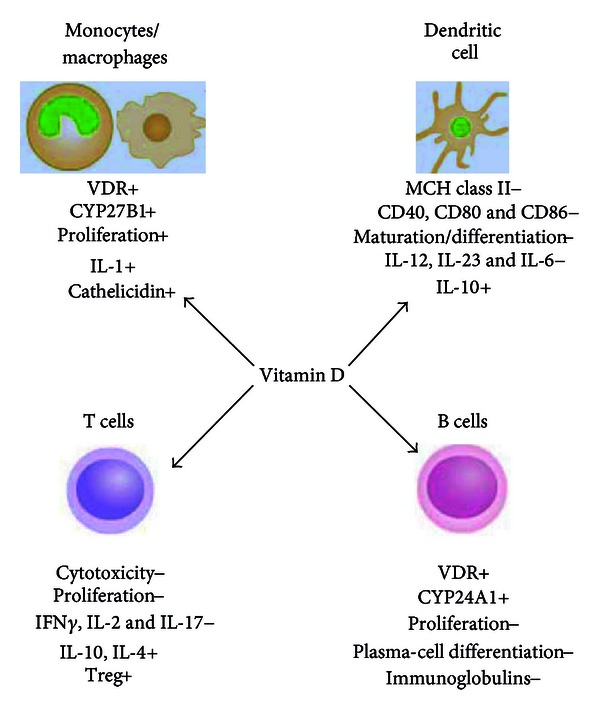
Effects of vitamin D on different immune system cells. Vitamin D regulates several immune system cell functions. It stimulates monocyte proliferation and differentiation towards macrophage-like cells, by self-increasing VDR, CYP27B, and IL-1 expressions; this “fast-forward” autocrine mechanism seems to be the basis for the subsequent maturation into macrophages, which does not take place in vitamin D deficient conditions; macrophage specific surface antigen expression is also enhanced. Vitamin D prevents T cells from proliferation, maturation, and releasing Th1-type molecules, such as IFN*γ*, IL-2, and IL-17, whereas it promotes Treg development. Vitamin D treatment prevents DCs from maturation and differentiation as well, by MHC class II, costimulatory factors, and interleukin downregulation. Vitamin D-induced protolerogenic DCs seem to be the key event for suppressive effects on immune system cells. Downregulation of B cell proliferation and maturation seems to be an indirect consequence of the suppressive effect exerted by vitamin D on T cells and APC. (+) and (−) indicate induction or inhibition.

VDR ligands usually exert their antiproliferative, prodifferentiation, and immunomodulatory effects throughout the activation of VDR—either constitutively present or induced—in the majority of the immune cells [32, 33].  Figure 1 summarizes the effects of vitamin D in different immune cell types. One of the first evidence for the immunoregulatory role of vitamin D was proven by the vitamin D-induced differentiation of monocyte precursors into mature macrophages [34]; the VDR high expression in monocytes has been hypothesized to be responsible for an autocrine mechanism for cell maturation, which, in fact, is impaired by vitamin D deficiency [35]. Monocytes from blood mononuclear cells (PBMCs) are able to synthesize vitamin D under inflammatory stimuli, such as interferon (IFN)*γ* or bacterial antigens [36, 37]; macrophage inflammatory response is modulated by vitamin D throughout the regulation of the release of critical inflammatory mediators, such as cytokines and chemotactic cytokines or chemokines. In both monocytes and macrophages, vitamin D regulates its own effects by controlling VDR and CYP27B1 expression and activity; signaling throughout Toll-like receptors (TLRs) is also engaged in association with VDR expression increase. In human monocytes treated with vitamin D, the expression of TLR2, TLR4, and TLR9 is inhibited, and TLR9-dependent interleukin (IL)-6 secretion is altered [38]. The observation that vitamin D, while promoting antimicrobial activity in myeloid cells, also inhibits TLR2 and TLR4 expressions in monocytes, suggested a feedback mechanism to prevent inflammatory overresponses by TLR activation at later stage of infection [39]; this downregulatory effect in APC might be one of the key mechanisms by which vitamin D is able to attenuate excessive Th1-driven inflammation and avoid downstream potential autoimmunity consequence [40]. Some stimulatory effects have been also shown on innate immunity, such as the increase of monocyte proliferation *in vitro *or IL-1 and cathelicidin (a bactericidal peptide) release by monocytes and macrophages [41, 42]; however, vitamin D effects on the adaptive immune response are predominantly suppressive.

In T cells, vitamin D inhibits not only proliferation but also IL-2 and IFN*γ* gene and protein expressions [43–46], likely through VDR-RXR complex interaction with VDREs in the promoter of the genes [47, 48]; it inhibits IL-17 and IL-2 expressions in CD4^+^ T cells and decreases CD8^+^ T cell-mediated cytotoxicity [49], with an overall effect towards a block of Th1-mediated response. Th2-type tolerogenic response is also promoted by a direct enhancement of IL-4 production [4]. Although vitamin D is known to stimulate the development and differentiation of regulatory T cells (Treg) enhancing their suppressive function [50–52], the direct effect on T cell differentiation and function is still unknown, since naïve T cells—differently from effector/memory T cells—express VDR at very low level [42]. However, it is quite clear that Treg cell differentiation is a key event connecting vitamin D with adaptive immunity, with potential beneficial effects for autoimmune diseases and host-graft rejection [3, 42, 53, 54]. It is widely accepted that those immunosuppressive functions are substantially driven by vitamin D induction of tolerogenic DCs [54–56]. In DCs, vitamin D inhibits differentiation and function as well, throughout a decrease in the expression of major histocompatibility complex (MHC) class II molecules and CD40, CD80, and CD86 [4, 57–59] costimulatory proteins; it decreases IL-6, IL-23, and IL-12 [60] while simultaneously increases IL-10 production. Those events also mirror a net decrease in Th1 cell response in favor of Th2-mediated events. By reducing IL-6 and IL-23 production, vitamin D likely inhibits also Th17 cells, another T cell subset deeply engaged in inflammatory responses; although the precise mechanism of vitamin D on Th17 regulation is still unclear [3], it seems that vitamin D-mediated Th1 and Th17 suppression occurs throughout Forkhead box protein 3 (Foxp3^+^) Treg cells expansion [3]. Furthermore, B cell proliferation, plasma-cell differentiation, and immunoglobulin (IgG) secretion are also affected by VDR ligands [1, 61], maybe throughout their effect on APC or T cells [62]. Vitamin D is likely to play a pivotal role in the maintenance of B cell homeostasis by regulating autoantibody production; notably, the correction of vitamin D deficiency might ameliorate B cell-mediated autoimmune disorders [63]. Finally, it is likely that endogenous production of vitamin D by macrophage, DCs, and T cells physiologically regulates both innate and adaptive immune responses [64–67]. Immune cells, indeed, seem to be not simple targets of VDR agonists but responsible for activation/inactivation of vitamin D metabolites [68].

### 3.2. Organ/Tissue Resident Cells

The ability of VDR agonists to modify the function of T cells and DCs depends not only on VDR expression in both cell types, but also on the presence of common targets in their signal transduction pathways, such as the nuclear factor *κ*B (NF-*κ*B) [69]. NF-*κ*B is a transcription factor well known to play a pivotal role in proinflammatory cytokine and chemokine production and release not just by immune system cells [70] but, remarkably, by different tissue resident cells, as previously reported [7–10, 12]. Based on VDR agonist capacity to inhibit NF-*κ*B activation in tissue resident cells, a strong reduction in local release of potent chemotactic factors by organ/tissue cells occurs; this, in turn, mirrors a reduced recruitment of Th1 cells, macrophages, and DCs to the site of inflammation. That feature of VDR agonists is particularly relevant for the treatment of inflammation involved in both auto- or alloimmune response, since it counteracts the mechanisms underlying the self-enhancing inflammatory loop between immune and resident cells. The potential therapeutic application of the less-hypercalcemic VDR agonist BXL-219 for autoimmune type 1 diabetes (T1D) has been highlighted in nonobese diabetic (NOD) mice, which retain a pathogenesis similar to the human disease. In this model, BXL-219-induced block of NF-*κ*Bp65 nuclear translocation is associated with decreased CXCL10 production by pancreatic islets, even in presence of restimulation with TLR agonists; this is reflected in a significant decrease in Th1 cell organ infiltration [71]. Similarly, we have reported that elocalcitol or BXL-628, a nonhypercalcemic VDR agonist, impairs NF-*κ*Bp65 and STAT1 nuclear translocation directly in human thyrocytes in association with a significant decrease in cytokine-induced CXCL10 release. This effect, in addition to a decreased Th1- and Th17-cytokine secretion by CD4^+^ T cells, makes elocalcitol to be a potential pharmacological tool in the treatment of autoimmune thyroid diseases [5]. 

## 4. CXCL10

Chemokines and their receptors are so far critical during inflammation to become novel targets for immunointervention [72, 73].

Among chemokines, CXCL10 plays a critical role in the initiation and maintenance of Th1-polarized response in autoimmune diseases or in graft injury; it appears to be directly linked to the disease pathogenesis and not related to a generic inflammatory status [11]. CXCL10 belongs to CXC chemokine subfamily and modulates innate and adaptive immune responses by controlling leukocyte trafficking [11, 74]. Under proinflammatory conditions, CXCL10 is secreted by several types of immune cells and by different resident cell types, as well [11]. CXCL10 exerts its action by binding the receptor CXCR3. Remarkably, local tissue secretion of CXCL10 represents the driving force for the recruitment of cytotoxic immune CXCR3-positive cells, such as T, natural killer (NK), B cells, macrophages, and DCs [75–77]. In particular, subtype A receptor activation leads to a potent CXCL10-induced chemotaxis for Th1 cell recruitment into inflammation sites [78], while the activation of subtype B, selectively expressed in human microvascular endothelial cells, is essentially involved in angiogenesis inhibition [79]. 

**Figure 2:**
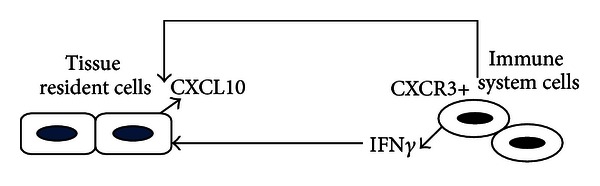
CXCL10-CXCR3 axis. CXCL10 secreted by different types of organ-resident cells under IFN*γ* induction is a potent chemoattractant for CXCR3-positive immune cells, in particular for activated T cells. T cell recruitment to sites of inflammation leads to an enhanced local increase of IFN*γ*, which, in turn, stimulates CXCL10 secretion by tissue cells; thus, a self-promoting inflammatory loop is established between resident and immune cells, making CXCL10-CXCR3 axis a therapeutic target for resolution of inflammation.

Hence, local CXCL10 production in inflammation sites is responsible for a positive feedback loop between IFN*γ*-producing Th1 cells and resident cells that, in turn, release CXCL10 upon IFN*γ* stimulation, as summarized in Figure 2 [80]. By those mechanisms a dominance of Th1-type cytokines and inflammatory response occurs together with a simultaneous Th2-type response downregulation.

The induction of CXCL10, important to protect against bacteria and some viruses infections, is described to be associated with inflammation processes engaged either in allo- or autoimmune response. Concerning the latter ones, different autoimmune diseases, such as rheumatoid arthritis (RA), systemic lupus erythematosus (SLE), systemic sclerosis (SSc), multiple sclerosis (MS), autoimmune thyroid diseases, Addison's disease, and T1D, are associated with an enhanced tissue expression of CXCL10, not only with increased circulating levels [81–85].

CXCL10-CXCR3 axis plays a pivotal role in the pathogenesis of graft failure and organ rejection, as well. In fact, CXCL10 is critical in promoting and amplifying host alloresponses responsible for acute allograft rejection [86–91]: CXCL10- or CXCR3-gene-deficient mice show permanent engraftment of cardiac transplants [86, 92]. In cardiac and small bowel models of allograft rejection, CXCL10 neutralization with monoclonal antibodies prolongs the allograft survival [86, 87]. CXCL10 intragraft expression level is associated with human renal [79], lung [89], and cardiac [90, 91] allograft rejections. In addition, intragraft CXCL10 expression correlates with damage degree, rejection, and even loss of the organ [11]. 

Circulating levels of CXCL10 are also increased and associated with the rejection rate in human recipients undergoing transplantation of organs (such as kidney, heart, lung, and liver), cardiopulmonary bypass, and allogeneic stem cell transplantation (SCT) [11, 93]. Importantly, CXCL10 high pretransplant serum levels may predict the risk for the development of acute rejection and chronic allograft vasculopathy (CAV) in different human organ rejection settings [11]. In particular, pretransplant CXCL10 serum assessment may be helpful in the prospective determination of the use of immune suppression therapy, in both renal and heart transplantations [11].

## 5. VDR Agonists and CXCL10

The protolerogenic properties of VDR agonists, as discussed above, render them suitable candidates as immunosuppressants for either autoimmune diseases or graft rejection, as clearly summarized by Mathieu and Adorini, since quite ago [94]. The *in vivo* effect of some VDR agonists on inflammatory mediators/processes involved in different diseases is depicted in Table 2 [95–102].

VDR agonists, besides their ability to switch the immune system cell balance from Th1 to Th2 dominance, are able to counteract CXCL10 production and release by several resident cell types [5–7]. Indeed, as previously mentioned, VDR ligands can prevent CXCL10 release by human thyrocytes or murine pancreatic cells, with potential benefits in autoimmune diabetes or thyroiditis [5, 53]. Those data are particularly intriguing in the light of the results from epidemiologic studies which underline an inverse correlation observed between vitamin D level and some autoimmune diseases, as SLE, RA, MS or SSc, in which chemokines seem to be engaged [103]; in addition, the vitamin D intake in early life of animal models prone to common autoimmune disorders (RA, MS, and autoimmune prostatitis) successfully prevents disease occurrence [4, 96, 104]; similarly, vitamin D supplementation in early childhood seems to be able to protect against T1D development [105]; accordingly, vitamin D serum levels are often decreased in patients with T1D [106], and subjects with a vitamin D deficiency are predisposed toward developing the disease [4, 107]. 

It is noteworthy that VDR agonist beneficial effects have been shown in different models of experimental organ transplantation as well—heart [98, 108–110], kidney [111, 112], liver [113, 114], pancreatic islets [71, 115–117], skin [99], and small bowel allografts [98]—since they delay acute and chronic allograft rejection. The latter effect, probably the most interesting in terms of potential clinical application, involves also the reduction of vascular intimal thickening—for example, due to vascular smooth muscle and endothelial cells hyperplasia—in association with a lesser extent of immune cell infiltration, after the treatment with VDR agonists [118]. Remarkably, many of the immunoregulatory properties of VDR agonists favorable in acute and chronic allograft rejection likely rely on their capacity to inhibit CXCL10 production by organ target cells, that is, *β* cells in mouse model of pancreatic islet transplantation [54] or human cardiomyocytes and renal tubular cells [6, 7] in heart or kidney transplantation. Of interest, in our hands, elocalcitol and BXL-01-0029 significantly decreased CXCL10 secretion, without cytotoxic effects neither in resident nor immune cells, differently from the majority of current immunosuppressants. Indeed, both VDR ligands left unchanged cardiac, renal, and CD4^+^ T cell viability, acting specifically on CXCL10 release [6, 7]. This effect is in line with the concept that vitamin D *in vivo* plays a pivotal role in immune homeostasis maintenance without strict immunosuppressants effects [68].

This observation could be of particular relevance as VDR agonists might serve as dose-reducing agents to add to conventional immunosuppressants in organ rejection management or autoimmune disease. Vitamin D analogues successfully decreased the doses of conventional immunosuppressive drugs in experimental autoimmune encephalomyelitis (EAE) model [4]; furthermore, in EAE, the addition of the vitamin D analogue TX527, with reduced calcemic activity, empowered the protective effect of IFN-*β* and CsA regimens, suggesting that this compound could be considered for clinical intervention in MS [102]; it is of interest that an association between CXCL10 and subjects affected with MS has been previously shown [119]. 

In organ transplantation, additive or synergistic effects are reported with VDR ligands and cyclosporine A (CsA), tacrolimus (FK-506) and sirolimus [120]. The combination of low-dose CsA with VDR agonists results in a significant decrease of IL-2 and IL-12 expressions and increased IL-10 in kidney allografts [112], likely by a reduction of renal bioactive transforming growth factor (TGF)-*β*. Furthermore, paricalcitol, a VDR activator, combined with trandolapril, an angiotensin-converting enzyme inhibitor, ameliorates obstructive nephropathy in a mouse model [121]. In humans, a retrospective study reports that vitamin D administration is able to delay renal graft loss in patients receiving conventional immunosuppressive drugs [122]. In isolated human tubular renal cells, the addition of BXL-01-0029 allows to lower FK-506 doses to reach the same inhibitory effect on cytokine-induced CXCL10 secretion [7]. 

## 6. Remarks and Conclusions

Given the pleiotropic effects of VDR agonists, based on their multifaceted interaction with immune and resident cells, it seems mandatory to encourage the research on those molecules, which, in light of their properties—due to their features to balance immune system homeostasis without being classical “immunosuppressants”—appear optimal candidates as novel therapeutic agents for Th1-driven inflammatory disease resolution. Large placebo-controlled, randomized-controlled trials should be encouraged since some discrepancies have been reported between *in vivo* and *in vitro* effects of vitamin D and its analogues, depending on both chemical structure (side chain configuration) and target cells [123]. Many clinical trials have been or are currently conducted by several investigators to test the therapeutic application of vitamin D or its derivatives in inflammatory processes underlying different pathologic conditions and diseases, that is, metabolic and/or kidney diseases (ClinicalTrial.gov, NCT01752244 and NCT00656032), inflammatory bowel diseases (Crohn's disease or ulcerative colitis, ClinicalTrial.gov NCT00122184 and NCT01426724), musculoskeletal diseases (ClinicalTrial.gov NCT01417923 and NCT01400009), and cardiovascular diseases (ClinicalTrial.gov NCT01331317). In this scenario, it is of particular interest a clinical trial evaluating vitamin D repletion, inflammation, and CXCL10 (ClinicalTrial.gov NCT01570309) in coronary artery disease.

Notably, VDR ligands are able to block CXCL10, that is a potential biomarker to monitor the inflammatory status but, more importantly for the topic of this paper, it represents a novel therapeutic target by which; that is, it could be feasible to fine-tune therapy for patients undergoing organ transplantation [11]. 

Furthermore, another benefit associated with the addition of VDR ligands to standard immunosuppressive regimens is related to their protective effects on bone loss [124]. Indeed, immunosuppressive agents are often associated with detrimental effects on bone. Together with bisphosphonates, vitamin D metabolites are the more extensively used molecules for bone-loss treatment. At variance with the first ones [118, 125], vitamin D analogues are indicated also in patients with adynamic bone disease. 

Finally, it is known since long time ago that vitamin D does not significantly interfere with protective immune response against infective pathogens [126] and displays antineoplastic properties [127], both quite relevant benefits in order to avoid opportunistic infection or tumor development, often associated with immunosuppression.

Despite the many advantages so far summarized, the use of VDR agonists in clinics is until now limited to calcipotriol, a vitamin D analogue topically applied for the treatment of psoriasis [128].

The limit in therapeutic applications of vitamin D undoubtedly relies on the systemic toxicity associated with long-term intake of this hormone; in fact, the supraphysiological doses of vitamin D necessary to reach the low local effective concentration (about 10^−10^ M) are associated with the undesirable risk of hypercalcemia [3, 129]. Therefore, the introduction of new molecules with immunosuppressive features without causing significant hypercalcemia has been strongly encouraged since a while [130]. Actually, drug development efforts should keep on designing vitamin D analogues retaining further distinct separation between immunomodulatory and hypercalcemic potency. Thus, the use of molecules as BXL-01-0029 or elocalcitol, with less or none hypercalcemic activity—and, therefore, without systemic toxicity—seems suitable for inclusion in immunosuppressive regimens, since they own the potentiality to lower the doses of current immunosuppressants and, thus, to reduce the side-effects associated with immunosuppression. 

## Figures and Tables

**Table 1:** VDR is almost ubiquitary expressed in humans. Many of human tissues and organs express VDR: upon ligand-receptor interaction genomic and nongenomic action likely occur by endocrine, paracrine, and autocrine mechanisms.

Human organs and tissues expressing vitamin D receptor (VDR)	
Adipose	Pancreatic *β*-cell
Adrenal	Parathyroid
Bone	Parotid
Brain	Pituitary
Breast	Placenta
Cartilage	Prostate
Colon	Retina
Hair follicle	Skin
Heart	Sperm
Intestine	Stomach
Kidney	Testis
Liver	Thymus
Lung	Thyroid
Immune cells	Tonsils
Muscle, smooth and skeletal	Uterus
Ovary	

**Table 2:** *In vivo* effect of some VDR agonists. Vitamin D analogs suppress inflammatory mediators and processes resulting in disease prevention.

Disease	Analogs	Main in vivo effects	Reference
Type 1 diabetes	KH1060	Type I diabetes prevention without significant effects on calcium or bone metabolism	[95]
Autoimmune prostatitis	BXL-628	Inhibition of the intraprostatic inflammatory response	[96]
Interstitial cystitis	BXL-628	Reduction of mast cell degranulation	[97]
Heart and small bowel graft	MC-1288	Delay/prevention of graft rejection	[98]
Skin allograft	KH1060, CB966	Skin allograft survival prolongation	[99]
Collagen-induced arthritis (CIA)	MC-1288	CIA prevention and suppression	[100]
Inflammatory bowel disease	TX527	Reduction in mucosal damage and crypt loss and suppression of the infiltration of immune cells	[101]
Experimental autoimmune encephalomyelitis (EAE)	TX527	EAE prevention	[102]
